# Evaluation of the Patients with the Diagnosis of Pontocerebellar Hypoplasia: A Multicenter National Study

**DOI:** 10.1007/s12311-024-01690-1

**Published:** 2024-04-15

**Authors:** Dilek Cavusoglu, Gulten Ozturk, Dilsad Turkdogan, Semra Hiz Kurul, Uluc Yis, Mustafa Komur, Faruk Incecik, Bulent Kara, Turkan Sahin, Olcay Unver, Cengiz Dilber, Gulen Gul Mert, Cagatay Gunay, Gamze Sarikaya Uzan, Ozlem Ersoy, Yavuz Oktay, Serdar Mermer, Gokcen Oz Tuncer, Olcay Gungor, Gul Demet Kaya Ozcora, Ugur Gumus, Ozlem Sezer, Gokhan Ozan Cetin, Fatma Demir, Arzu Yilmaz, Gurkan Gurbuz, Meral Topcu, Haluk Topaloglu, Ahmet Cevdet Ceylan, Serdar Ceylaner, Joseph G. Gleeson, Dilara Fusun Icagasioglu, F. Mujgan Sonmez

**Affiliations:** 1 Departments of Pediatric Neurology, Afyonkarahisar Health Sciences University, Afyon, Turkey; 2 Departments of Pediatric Neurology, Marmara University, Istanbul, Turkey; 3 Departments of Pediatric Neurology, Dokuz Eylul University, Izmir, Turkey; 4 Departments of Pediatric Neurology, Mersin University, Mersin, Turkey; 5 Departments of Pediatric Neurology, Cukurova University, Adana, Turkey; 6 Departments of Pediatric Neurology, Kocaeli University, Kocaeli, Turkey; 7 Departments of Pediatric Neurology, Bezmialem Vakif University, Istanbul, Turkey; 8 Departments of Pediatric Neurology, Kahramanmaras Sutcu Imam University, Kahramanmaras, Turkey; 9 Izmir International Biomedicine and Genome Institute, Dokuz Eylül University, Izmir, Turkey; 10 Departments of Medical Genetics, Mersin University, Mersin, Turkey; 11 Departments of Pediatric Neurology, Ondokuz Mayıs University, Samsun, Turkey; 12 Departments of Pediatric Neurology, Pamukkale University, Denizli, Turkey; 13 Departments of Pediatric Neurology, Hasan Kalyoncu University, Gaziantep, Turkey; 14 Departments of Medical Genetics, Dr Ersin Arslan Training and Research Hospital, Gaziantep, Turkey; 15 Departments of Medical Genetics, Samsun Training and Research Hospital, Samsun, Turkey; 16 Departments of Medical Genetics, Pamukkale University, Denizli, Turkey; 17 Departments of Medical Genetics, Ankara Bilkent City Hospital, Ankara, Turkey; 18 Departments of Pediatric Neurology, Ankara Training and Research Hospital, Ankara, Turkey; 19 Departments of Pediatric Neurology, Tekirdag Namik Kemal University, Tekirdag, Turkey; 20 Departments of Pediatric Neurology, Hacettepe University,Retired Lecturer, Ankara, Turkey; 21 Departments of Pediatric Neurology, Yeditepe University, Istanbul, Turkey; 22 Intergen Genetic Research Center, Ankara, Turkey; 23 Department of Neurosciences and Pediatrics, Rady Children’s Institute for Genomic Medicine, Howard Hughes Medical Institute, University of California, La Jolla, San Diego, CA USA; 24 Departments of Pediatric Neurology, Department of Child Neurology, Karadeniz Technical University Medical Faculty, Retired Lecturer, Trabzon, Turkey; 25 Yuksek Ihtisas University, Faculty of Medicine, Ankara, Turkey; 26 Aziziye Mah. Cinnah Cad. 102/3, Cankaya, Ankara, Türkiye

**Keywords:** Pontocerebellar Hypoplasia, *CLP1*, Genotype, Phenotype

## Abstract

Pontocerebellar hypoplasia (PCH) is a heterogeneous group of neurodegenerative disorders characterized by hypoplasia and degeneration of the cerebellum and pons. We aimed to identify the clinical, laboratory, and imaging findings of the patients with diagnosed PCH with confirmed genetic analysis. We collected available clinical data, laboratory, and imaging findings in our retrospective multicenter national study of 64 patients with PCH in Turkey. The genetic analysis included the whole-exome sequencing (WES), targeted next-generation sequencing (NGS), or single gene analysis. Sixty-four patients with PCH were 28 female (43.8%) and 36 (56.3%) male. The patients revealed homozygous mutation in 89.1%, consanguinity in 79.7%, pregnancy at term in 85.2%, microcephaly in 91.3%, psychomotor retardation in 98.4%, abnormal neurological findings in 100%, seizure in 63.8%, normal biochemistry and metabolic investigations in 92.2%, and dysmorphic findings in 51.2%. The missense mutation was found to be the most common variant type in all patients with PCH. It was detected as *CLP1* (*n* = 17) was the most common PCH related gene. The homozygous missense variant c.419G > A (p.Arg140His) was identified in all patients with *CLP1.* Moreover, all patients showed the same homozygous missense variant c.919G > T (p.A307S) in *TSEN54* group (*n* = 6). In Turkey, *CLP1* was identified as the most common causative gene with the identical variant c.419G > A; p.Arg140His. The current study supports that genotype data on PCH leads to phenotypic variability over a wide phenotypic spectrum.

**Supplementary Information:**

The online version contains supplementary material available at 10.1007/s12311-024-01690-1.

## Introduction

Pontocerebellar hypoplasia (PCH) demonstrates a group of heterogeneous neurodegenerative disorders with concurrent hypoplasia of the pons and the cerebellum and also variable clinical and neuroimaging findings including supratentorial involvement [1–3]. The first case of PCH was reported in 1917 [4]. Barth et al. described seven children with PCH from five families [5]. The clinical findings occurred of involuntary movements, extrapyramidal dyskinesia, and microcephaly. Moreover, Barth proposed the first classification including PCH1 and PCH2 subtypes. PCH1 showed hypotonia and muscle weakness depending on anterior horn cell degeneration. PCH2 is separated by jitteriness in the neonatal period, poor sucking and swallowing, chorea/dyskinesia, and profound neurodevelopmental and cognitive delay [6].

The current classification (OMIM, Online Mendelian Inheritance in Man) identified 17 subtypes and 25 genes of PCH attributed to clinical, neuroradiological, and biochemical features, and gene analysis. The clinical spectrum has been expanded to different neurological phenotypes. Clinical manifestations consist of global developmental delay and variable neurological features. All subtypes represent autosomal recessive inheritance. Neuroimaging patterns containing infratentorial and supratentorial anomalies in addition to reduced volume of pons and cerebellum may have diagnostic value in some subtypes of PCH [7]. Some genotype–phenotype correlation has been described even after PCH was identified. One of these, homozygosity for the p.A307S mutation in *TSEN54* with PCH2A (OMIM # 277470) presents progressive microcephaly, spasticity, poor swallowing, central visual defect with the absence of primary optic atrophy, dyskinesia and/or dystonia, hypertonia at birth with dragonfly-like cerebellum with flattened cerebellar hemispheres and a relatively preserved vermis [1, 3, 7]. Moreover, dragonfly appearance of the cerebellum is often observed in PCH2 and PCH4[8]. It is also suggested that a more severe clinical presentation including respiratory failure, polyhydramnios, contractures and early death, is associated with nonsense or splice site mutations in *TSEN54* [3]. Pathogenic mutations in *EXOSC3* with PCH 1B (OMIM # 614678) are described by severe muscle weakness, axial hypotonia, spasticity, tongue fasciculations, contractures, marked psychomotor retardation, and axonal motor neuropathy similar to spinal muscular atrophy (SMA) [1, 2, 7]. Neuroradiological findings of PCH1 may show moderate hypoplasia and atrophy of the pons and cerebellum [8]. Recently some reports have suggested the question regarding the genotype–phenotype relationship in *EXOSC3* due to the identification of the milder phenotypes [9]. Although broad mutation spectrum of the *CASK* gene, the phenotype shows facial dysmorphism, microcephaly (especially < -3 SD occipitofrontal circumference), hearing impairment, optic atrophy, retinopathy, hypohidrosis, developmental delay, limb hypertonia, pronounced cerebellar hypoplasia, diverse degrees of pons hypoplasia, and a normal-sized corpus callosum [7]. PCH3 related to a homozygous mutation in the *PCLO* gene is defined associated with optic atrophy and thin corpus callosum on MRI. The typical imaging findings of *AMPD2* related to PCH9 are characterized by a dragonfly-like cerebellum and figure of “8” on axial images consisting of a thin corpus callosum and abnormal midbrain. PCH9 also exhibits hypodysgenesis of corpus callosum [8].

*CASK*- and *VLDLR*-associated disorders, uncommon variants associated with *DCK1, WDR81, ITPR1* gene mutations, some congenital disorders of glycosylation and tubulinopathies, and particular dystroglycanopathies are also reported related to PCH [7].

We aim to investigate the clinical, laboratory, and neuroimaging findings with the diagnosis of PCH confirmed by genetic analysis by a multicenter study in Turkey.

## Materials and Methods

We retrospectively collected the comprehensive clinical data of 64 patients with diagnosed PCH confirmed by genetic analysis from 15 different neurological centers and seven geographical regions in Turkey. Age of diagnosis, gender, consanguinity, pregnancy duration, occipital frontal circumference (OFC) at the examination, psychomotor development, history of seizure, dysmorphic and neurological findings, neuroimaging features, other system findings, biochemistry and metabolic tests including including urine and blood aminoacid, tandem MS + acycl carnitine, urine organic acids, very long chain fatthy acids, biotinidase activity, transferrin focusing, and, genetic analysis of the patients were evaluated. Microcephaly is desciribed as OFC is two or more standard deviations (SD) lower than the mean for age and gender. The genetic analysis comprised the whole-exome sequencing (WES), targeted next-generation sequencing (NGS) or single gene analysis.

The findings of brain magnetic resonance imaging were collected. The infratentorial and supratentorial involvement were enrolled. The presence of ventriculomegaly, abnormalities of cerebral cortex (atrophy/thicking), white matter, and corpus callosum were interpreted in addition to pontocerebellarr hypoplasia. Furthermore, the patiens with a particular patter such as dragonfly pattern (comparatively preserved vermis and atrophic cerebellar hemispheres), butterfly pattern (a small, normally proportioned cerebellum) and figure of “8” of the midbrain were recorded.

Patients number 14 and 17[10], 26 and 27[11], 28–31[12], 39–42[13–15] and 60, 61[15] from previously published article were included in the study (supplementary material).

The approval of Bezmialem Vakıf University's ethics committee was obtained (approval number: E-54022451–050.05.04–70596/26.07.2022).

## Statistical Analysis

Statistical analysis was performed using Statistical Package for the Social Sciences (SPSS) software version 21.0 (SPSS Inc., Chicago, Ill., USA). Frequencies and percentages were calculated. Descriptive statistics were performed. Categorical variables were expressed in numbers, and continuous variables were summarized as mean and standard deviation (SD).

## Results

A total of 64 patients with PCH were included in the study from seven geographical regions in Turkey, 28 were female (43.8%) and 36 (56.3%) were male.

**Fig. 1:**
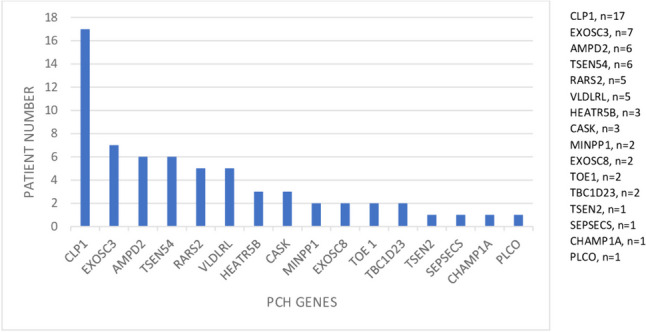
Distribution of PCH genes

**Fig. 2:**
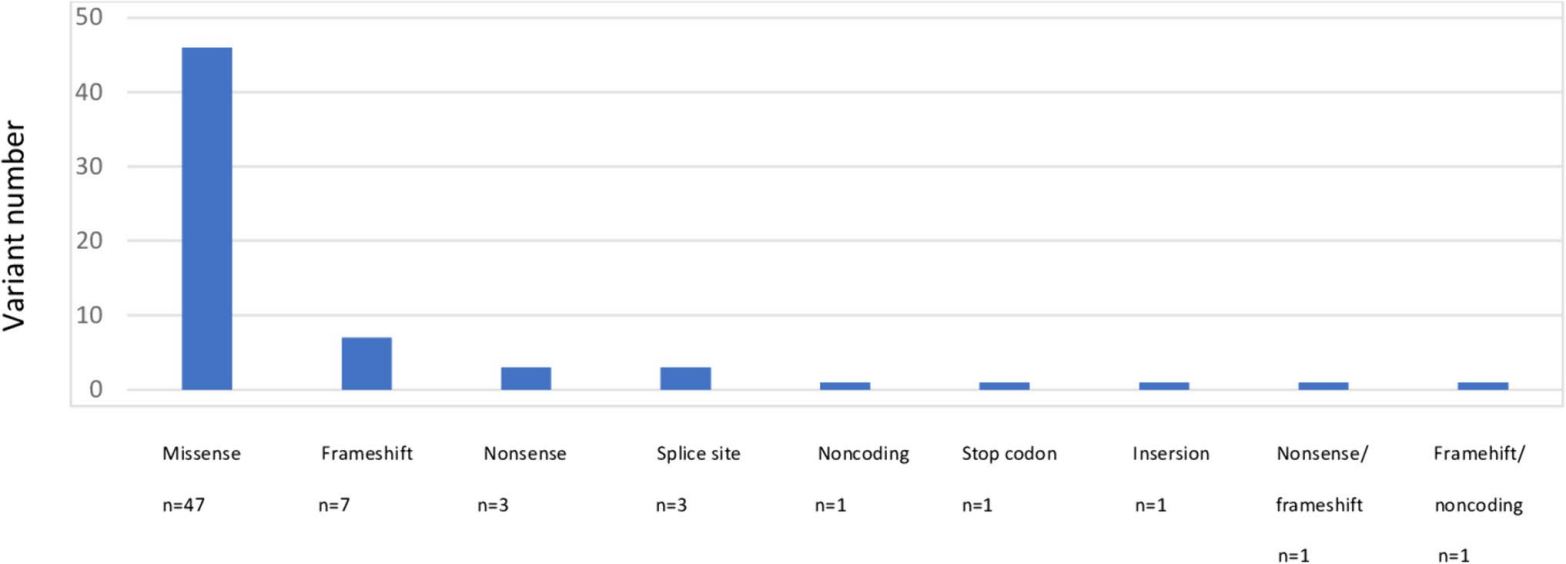
Variant type in PCH

We identified 16 distinct PCH types and PCH-related genes ( % and number): *EXOSC3* (PCH1B) ( 10.9%, *n =* 7), *EXOSC8* (PCH1C) (3.1%, *n =* 2), *TSEN2* (PCH2B) (1.5%, *n =* 1), *SEPSECS* (PCH2D)( 1.5%, *n =* 1), *PCLO* ( PCH3) (1.5%, *n =* 1), *TSEN54* (PCH5)(9.3%, *n =* 6), *RARS2* (PCH6) (7.8%, *n =* 5), *TOE1* (PCH7) (3.1%, *n =* 2), *CHMP1A* (PCH8)( 1.5%, *n =* 1), *AMPD2* (PCH9) (9.3%, *n =* 6), *TBC1D23* (PCH11) (3.1%, *n =* 2), *CLP1* (PCH10) (26.56%, *n =* 17), *MINPP1* (PCH16)( 3.1%, *n =* 2), *VLDLRL* (7.8%, *n =* 5), *HEATR5B* (4.6%, *n =* 3), and *CASK* (4.6%, *n =* 3) (Fig. 1). The most common associated gene was *CLP1* (26.56%). The distribution of patients with PCH in the geographical regions revealed as the Mediterranean (*n =* 20), Marmara (*n =* 15), Black sea (*n =* 11), aegean (*n =* 8), inner Anatolian (*n =* 5), southeastern Anatolia (*n =* 3), and eastern Anatolia (*n =* 2). The confirmed genetic analysis occured of WES (*n =* 51), targeted next-generation sequencing (*n =* 10), and single gene analysis (*n* = 3). The most common variant type was detected as missense (*n =* 47) and followed by frame shift (*n =* 7), nonsense (*n =* 3) and splice site (*n =* 3), respectively (Fig. 2). The patients showed homozygous mutation in 89.1% (57/65).

Consanguinity was described in 79.7% (51/64), pregnancy at term in 85.2% (52/61), abnormal neurological findings including gros motor (delay/absent), fine motor (delay/absent)and intellectual disability(except for in *SEPSECS* group) were determined in 100% (64/64). Microcephaly (42/46), dysmorphic findings (22/43) and seizure (37/58) (except for *TSEN2, SEPSECS* and *PCLO* groups), were found in 91.3%, 51.2%, and 63.8%, respectively. Cardiovascular disorder including atrial septal defect, patent ductus arteriosus, and patent foramen ovale were seen in one patient in each of the following groups *CLP1, MINPP1,* and *CHMP1A*. In *TBC1D23* group (two patient) atrial septal defect was seen as a cardiac findings. Eye abnormalities ( poor eye contact, strabismus, microphthalmia, optic atrophy, cataract, ptosis, astigmatism, cortical visual impairment) were described in 69.8% (37/53). Musculoskeletal disorders such as scoliosis, muscle atrophy, hip dislocation, contractures, joint stiffness, kyphosis, genitourinary disorders including micropenis, undescended testes, abnormal genitalia, urinary incontinence, recurrent urinary infection gastrointestinal disorders such as constipation, stool incontinence, feeding abnormalities, feeding with percutaneous endoscopic gastrostomy tube, aganglionic megacolon, gallstone, endocrinological findings including hypothyroidism and hypocalcemia were determined in 56.8% (29/51), 28.1% (9/32), 50%(24/48), and 30.7%(4/13), respectively.

Immunodeficiency including recurrent infections were described one patient in each of *EXOSC3* and *RARS2* groups and two patient in *TSEN54* and *HEATR5B* groups. Brainstem findings (hyperacusis, central apnea, dizziness, vertigo, impaired swallowing) and cerebellar deficits (nystagmus, intentional tremor, dysmetria, scanning speech) were revealed in 55.3% (31/56) and 67.3% (31/46), respectively. Hypotonia (except for *VLDL, MINPP1, TOE1, TSEN2, SEPSECS, CHMP1A* and *PCLO*), hypertonia (except for *RARS2, HEATHR5, CASK, EXOSC8, TC1D23; TSEN2, CHMP1A* and *PCLO*) and axial hypotonia and distal hypertonia (AHDH) (*CLP1, EXOSC3, AMPD2, RARS2, CASK, MINPP1, CHMP1A* and *PCLO*) were determined in 38.9%(23/59), 30.5% (18/59), and 23.7% (14/59), respectively. Behavioral disorders including autism and ADHD (Attention deficit hyperactivity disorder) were described in 30.4% (14/46), and 22% (11/50), respectively.

**Fig. 3:**
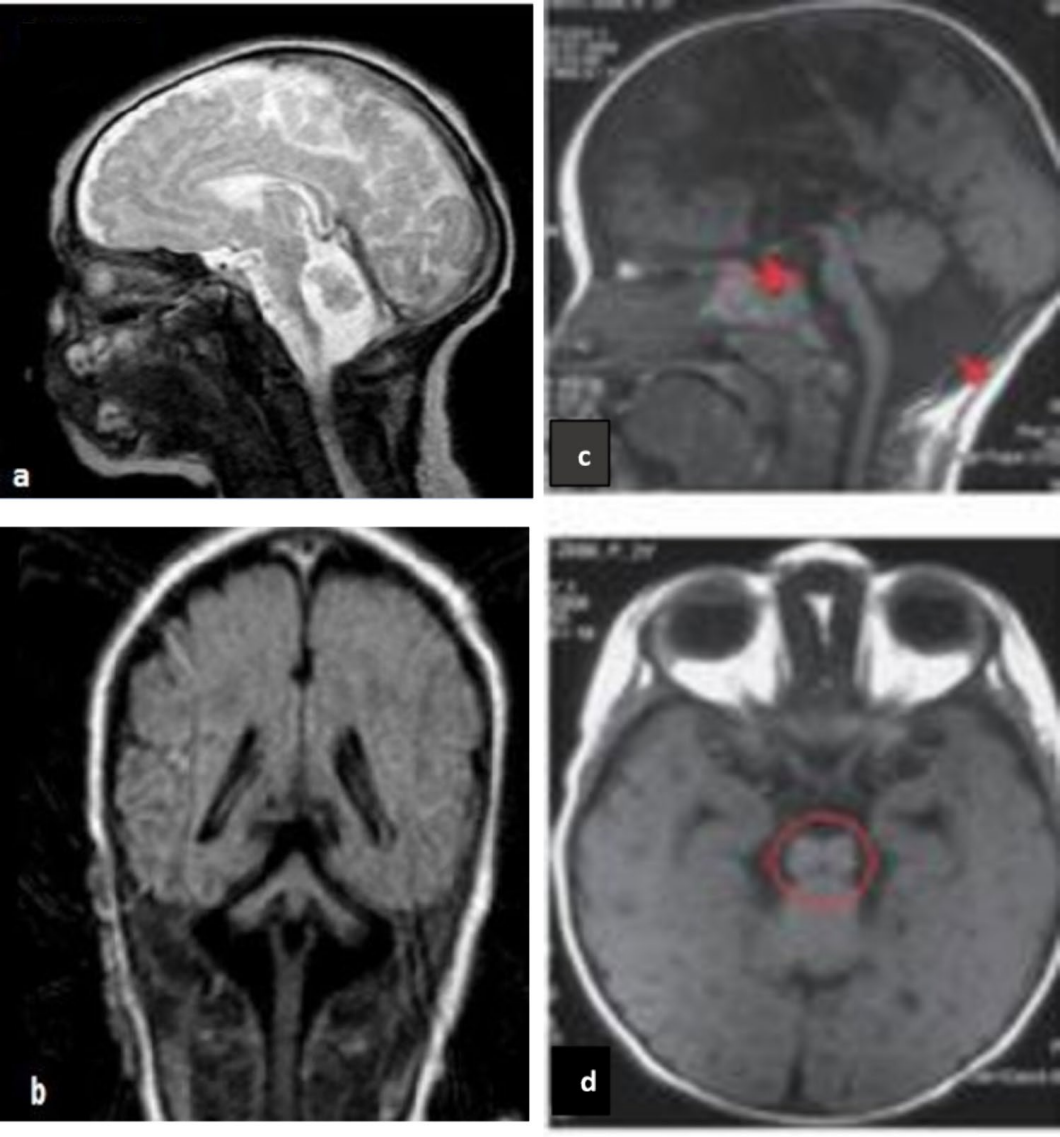
(a-d) Typical radiological findings shown—(a, b) dragonfly pattern (comparatively preserved vermis and atrophic cerebellar hemispheres) in one patient with TSEN54 gene. (c, d) brainstem appearance of figure of 8 on the brain MRI of one patient with AMPD2 gene

Biochemical and metabolic tests were normal in 92.2% (46/50). Biochemical and metaboic investigations showed high lactate level, anemia, polycythemia in two patients with *CLP1* and one patient in each of the *RARS2* and *HEATR5B* groups. Except for PCH appearence, MRI findings of the patients revealed the particular features such as the butterfly pattern in *EXOSC3* (*n =* 1), the dragonfly pattern in *AMPD2* (*n =* 1) and *TSEN54* (*n =* 2), the flattening pons in *CLP1* (*n =* 2) and a figure of “8” of the midbrain in *AMPD2* (*n =* 2) (Fig. 3). The detailed demographic, clinical, EEG and MRI findings of each gene group was summarised in Tables 1, 2 and 3.

**Table 1:** Demographic findings of PCH patients

GENE	CLP1	EXOSC3	AMPD2	TSEN54	RARS2	VLDLRL	HEATR5B	CASK	MINPP1	EXOSC8	TOE1	TBC1D23	TSEN2	SEPSECS	CHMP1A	PCLO
Patient	(*n* = 17)	(*n* = 7)	(*n* = 6)	(*n* = 6)	(*n* = 5)	(*n* = 5)	(*n* = 3)	(*n* = 3)	(*n* = 2)	(*n* = 2)	(*n* = 2)	(*n* = 2)	(*n* = 1)	(*n* = 1)	(*n* = 1)	(*n* = 1
Gender ( Male/Female)	11/6	5/2	2/4	3/3	3/2	3/2	1/2	1/2	1/1	1/1	1/1	2/0	0/1	0/1	1/0	1/0
Age of diagnosis (mean ± SD, year)	4.91 ± 3.65	7.76 ± 5.54	3.61 ± 2.46	7.5 ± 6.36	2.03 ± 1.86	15 ± 15.55	0.75	3.27 ± 1.11	2.83	8.5 ± 6.36	5 ± 0.7	5 ± 1.41	1.91	2.09	3	10
Age at last FU(mean ± SD,year)	6.61 ± 4.82	9.39 ± 4.74	5.93 ± 1.85	6.83 ± 4.07	3.96 ± 2.92	14 ± 4.24	2.2 ± 1.12	3.88 ± 1.17	2.83	9.5 ± 4.94	6 ± 0.7	NA	1.91	2.09	4	11
Term pregnancy (%)	16/16(100)	4/7(57.1)	6/6(100)	6/6(100)	4/5(80)	3/4(75)	2/3(66.6)	3/3(100)	1/1(100)	1/2(50)	2/2(100)	2/2(100)	1/1(100)	1/1(100)	-	-
Consanguinity (%)	14/17(82.3)	5/7(71.4)	5/6(83.3)	5/6(83.3)	4/5(80)	5/5(100)	3/3(100)	1/3(33.3)	2/2(100)	1/2(50)	2/2(100)	2/2(100)	1/1(100)	-	1/1(100)	-

*FU* follow-up

**Table 2:** Clinical findings of PCH patients

GENE	CLP1	EXOSC3	AMPD2	TSEN54	RARS2	VLDLRL	HEATR5B	CASK	MINPP1	EXOSC8	TOE1	TBC1D23	TSEN2	SEPSECS	CHMP1A	PCLO
Patient	(*n* = 17)	(*n* = 7)	(*n* = 6)	(*n* = 6)	(*n* = 5)	(*n* = 5)	(*n* = 3)	(*n* = 3)	(*n* = 2)	(*n* = 2)	(*n* = 2)	(*n* = 2)	(*n* = 1)	(*n* = 1)	(*n* = 1)	(*n* = 1)
Microcephaly at last FU (%)	11/11(100)	2/5(40)	3/3(100)	3/3(100)	3/4(75)	4/4(100)	2/2(100)	3/3(100)	1/1(100)	2/2(100)	2/2(100)	2/2(100)	1/1(100)	1/1(100)	1/1(100)	1/1(100)
Development delay (%)	17/17(100)	7/7(100)	6/6(100)	6/6(100)	5/5(100)	5/5(100)	3/3(100)	3/3(100)	1/2(50)	2/2(100)	2/2(100)	2/2(100)	1/1(100)	1/1(100)	1/1(100)	1/1(100)
Regression (%)	6/17(35.2)	2/7(28.5)	-	1/6(16.6)	1/5(20)	-	3/3(100)	-	1/2(50)	-	2/2(100)	-	-	-	1/1(100)	-
Intellectual disability (%)	17/17(100)	7/7(100)	6/6(100)	6/6(100)	5/5(100)	5/5(100)	3/3(100)	3/3(100)	NA	2/2(100)	2/2(100)	2/2(100)	1/1(100)	-	1/1(100)	1/1(100)
Autistic features (%)	6/13(46.1)	1/4(25)	1/5(20)	-	2/3(66.6)	-	NA	1/2(50)	NA	2/2(100)	-	1/2(50)	-	-	NA	-
ADHD (%)	3/15(20)	3/7(42.8)	-	-	-	-	NA	1/2(50)	NA	1/2(50)	-	2/2(100)	1/1(100)	-	NA	-
Seizure (%)	12/17(70.5)	2/5(40)	4/6(66.6)	4/5(80)	5/5(100)	-	2/2(100)	1/3(33.3)	1/1(100)	2/2(100)	1/2(50)	2/2(100)	-	-	1/1(100)	-
-Onset (mean ± SD,month)	12.66 ± 15.41	2	16.8 ± 14.18	9.8 ± 15.15	8.67 ± 13.27	-	1	1	2	6.5 ± 2.12	48	24 ± 16.97	-	-	-	-
- Frequency	5D 3Y	1Y	3D1Y	1D2M	4D	-	2D1Y	1D	NA	NA	1D	2Y	-	-	NA	-
-Type	6G/2F/1BA/ 2G + F	1G/ 1F + Mc	2G/2G + F/ 1EE	1G/1F/ 1F + Mc	2G/1F/ 2Mc	-	1G/1F/ 1G + F	1EE	NA	1G/1F	1G	2F	-	-	NA	-
-Seizure free patient number,( %)	3/11(27.2)	1/2(50)	-	1/4(25)	1/5(20)	-	-	-	-	1/1(100)	-	-	-	-	-	-
-Controlled seizure (%)	6/10(60)	1/1(100)	1/4(25)	1/4(25)	1/5(20)	-	NA	-	1/1(100)	1/1(100)	-	2/2(100)	-	-	-	-
-Monotheraphy/Polytheraphy	6Mo/5Po	1Mo	1Mo/3Po	1Mo/3Po	1Mo/2Po	-	NA	1Po	1Mo	1Mo/1Po	1Po	2Mo	-	-	1Mo	-
Dysmorphic features (%)	9/15(60)	-	-	1/5(20)	2/4(50)	1/4(25)	-	2/2(100)	1/1(100)	1/2(50)	2/2(100)	2/2(100)	-	-	1/1(100)	-
Visual abnormality (%)	2/13(15.3)	1/2(50)	1/6(16.6)	-	1/3(33.3)	-	2/3(66.6)	1/1(100)	NA	1/2(50)	-	-	-	-	1/1(100)	NA
Brainstem findings (%)	8/16(50)	6/6(100)	2/5(40)	1/5(20)	2/5(40)	-	3/3(100)	2/3(66.6)	2/2(100)	1/1(100)	-	2/2(100)	-	-	1/1(100)	1/1(100)
Cerebellar deficit (%)	5/12(41.6)	6/6(100)	2/5(40)	-	2/3(66.6)	4/5(80)	2/3(66.6)	2/2(100)	2/2(100)	1/1(100)	2/2(100)	2/2(100)	NA	NA	1/1(100)	NA
Tetraplegia (%)	13/15(86.6)	2/6(33.3)	6/6(100)	4/6(66.6)	3/4(75)	-	1/1(100)	2/2(100)	1/1(100)	1/1(100)	1/2(50)	-	1/1(100)	-	1/1(100)	1/1(100)
Paraplegia (%)	2/15(13.3)	1/6(16.6)	-	2/6(33.3)	-	4/5(80)	-	-	-	-	1/2(50)	-	-	1/1(100)	-	-
Hypotonia (%)	7/17(41.1)	2/6(33.3)	2/6(33.3)	1/6(16.6)	4/5(80)	-	3/3(100)	1/3(33.3)	-	1/1(100)	-	2/2(100)	-	-	-	-
Hypertonia (%)	6/17(35.2)	1/6(16.6)	1/6(16.6)	5/6(83.3)	-	1/2(50)	-	-	1/2(50)	-	2/2(100)	-	-	1/1(100)	-	-
AHDH (%)	4/17(23.5)	2/6(16.6)	3/6(50)	-	1/5(20)	-	-	1/3(33.3)	1/2(50)	-	-	-	-	-	1/1(100)	1/1(100)
Gait disorder (ataxia,spastic) (%)	14/15 (93.3)	5/5(100)	-	1/1(100)	3/3(100)	3/3(100)	1/1 (100)	2/3(66.6)	1/1(100)	2/2(100)	2/2(100)	2/2(100)	-	1/1(100)	1/1(100)	-

*ADHD* Attention deficit hyperactivity disorder, *AHDH* Axial hypotonia and distal hypertonia, *BA* Behavior arrest, *D* Daily, *EE* Epileptic encephalopathy, *G* Generalized, *F *Focal, *M* Monthly, *Mc* Myoclonic, *Mo* Monotheraphy, *NA * Not available, *Po* Polytheraphy, *Y* Yearly

**Table 3:** Other system findings, laboratory and neuroimaging features of PCH patients

GENE	CLP1	EXOSC3	AMPD2	TSEN54	RARS2	VLDLRL	HEATR5B	CASK	MINPP1	EXOSC8	TOE1	TBC1D23	TSEN2	SEPSECS	CHMP1A	PCLO
Patient	(*n* = 17)	(*n* = 7)	(*n* = 6)	(*n* = 6)	(*n* = 5)	(*n* = 5)	(*n* = 3)	(*n* = 3)	(*n* = 2)	(*n* = 2)	(*n* = 2)	(*n* = 2)	(*n* = 1)	(*n* = 1)	(*n* = 1)	(*n* = 1)
Eye abnormality (%)	8/15(53.3)	3/4(75)	2/5(40)	2/6(33.3)	5/5(100)	5/5(100)	2/2(100)	3/3(100)	2/2(100)	1/2(50)	2/2(100)	2/2(100)	-	-	NA	-
Immunodeficiency (%)	-	1/3(33.3)	-	2/6(33.3)	1/5(20)	-	2/3(66.6)	-	-	-	-	-	-	-	-	-
Cardiovascular disorder (%)	1/14(7.14)	-	-	-	-	-	-	-	1/1(100)	-	-	2/2(100)	-	-	1/1(100)	-
Respiratory disorder (%)	3/13(23)	-	-	1/5(20)	-	-	3/3(100)	1/3(33.3)	-	1/2(50)	-	-	-	-	-	-
Endocrine disorder (%)	-	1/5(20)	-	1/6(16.6)	-	-	-	-	-	-	-	2/2(100)	-	-	-	-
Musculoskeletal disorder (%)	11/17(64.7)	2/3(66.6)	3/6(50)	1/4(25)	1/5(20)	1/4(25)	-	2/3(66.6)	1/1(100)	1/2(50)	2/2(100)	2/2(100)	-	-	1/1(100)	1/1(100)
Genitourinary disorder (%)	3/14(21.4)	1/5(20)	2/5(40)	1/6(16.6)	-	-	-	-	1/1(100)	-	1/1(100)	-	-	-	-	-
Gastrointestinal disorder (%)	6/15(40)	4/6(66.6)	1/6(16.6)	1/5(20)	4/5(80)	-	3/3(100)	2/3(66.6)	-	1/2(50)	-	1/2(50)	-	-	-	1/1(100)
Biochemical and metabolic abnormality (%)	2/15(13.3)	-	-	-	1/4(25)	-	1/3(33.3)	-	-	-	-	-	-	-	-	-
EEG abnormality (%)	10/14(71.4)	2/5(40)	5/6(83.3)	3/5(60)	5/5(100)	-	3/3(100)	1/3(33.3)	1/2(50)	2/2(100)	1/1(100)	2/2(100)	-	-	1/1(100)	-
VEP/ERG abnormality (%)	2/4(50)	-	-	1/1(100)	1/1(100)	-	-	-	1/1(100)	-	-	1/2(50)	-	-	1/1(100)	-
ENMG abnormality (%)	4/7(57.1)	1/4(25)	-	-	-	-	-	-	-	1/2(50)	-	-	-	-	-	-
*Brain MRI																
Age at the MRI (mean ± SD, year)	3.69 ± 3.13	6.26 ± 5.24	5.77 ± 1.57	3.04 ± 3.51	2.3 ± 2.38	0.58	0.66	3.91 ± 3.64	NA	5.5 ± 3.53	2.25 ± 0.35	4.0 ± 1.41	NA	NA	2	8
Cerebral cortex																
-Thicking(%)	-	-	-	-	-	3/4(75)	-	-	-	-	-	-	-	-	-	-
-Atrophy(%)	11/17(64.7)	3/4(75)	5/6(83.3)	1/5(20)	3/5(60)	-	2/2(100)	-	1/1(100)	-	2/2(100)	-	-	-	-	-
Ventriculomegaly (%)	8/17(47)	1/1(100)	3/6(50)	2/5(40)	1/4(25)	-	3/3(100)	-	1/1(100)	-	2/2(100)	2/2(100)	-	-	1/1(100)	1/1(100)
White matter abnormality (%)	4/16(25)	2/3(66.6)	2/5(40)	1/6(16.6)	2/4(50)	-	2/2(100)	1/3(33.3)	2/2(100)	-	-	-	-	-	1/1(100)	-
Corpus callosum abnormality (%)	14/16(87.5)	3/3(100)	5/6(83.3)	3/5(60)	1/5(20)	-	3/3(100)	-	1/1(100)	-	2/2(100)	2/2(100)	-	-	1/1(100)	-

*NA* Not available, *VEP/ERG* visual evoked potential/electroretinography, *:Features except pontocerebellar hypoplasia in MRI

### CLP1

We determined that *CLP1* was the most common PCH form in Turkey. This group included eleven male and six female patients. Consanguinity was described in 82.3% of the patients. Term pregnancy, microcephaly, development delay(gross motor/fine motor/language/social),, and intellectual disability were observed in 100% of the patients. Dysmorphic features, musculoskeletal disorders (scoliosis, muscle atrophy, hip dislocation, contractures, joint stiffness, kyphosis), brainstem findings (central apnea, dizziness, vertigo, impaired swallowing) and cerebellar deficit ( nystagmus, intentional tremor, dysmetria, scanning speech) were determined 60%, 64.7%, 50%, 41.6%, respectively. Tetraplegia were described in 86.6% and paraplegia in 13.3%. Gait disorders including ataxia and/or spastic are reported in 14 out of 15 (93.3%) patients. Seizures were found in 70.5% of the patients. The onset of the seizures were described at 12.66 ± 15.41 months. Frequency of seizure were every day in five patients and once a year in three patients. Additionally, seizures were treated with monotheraphy in six patients and polytheraphy in 5 patients. Among the patients, seizures were controlled in six patients. Three of them were determined as seizure free. Regression, autistic features and ADHD were described 35.2%, 46.1%, and 20%, respectively. EEG abnormalities were identified 71.4% of the patients. All EMG findings were found to be compatible with axonal sensorimotor/motor neuropathy (*n =* 4). The age of the first MRI was 3.69 ± 3.13 years. MRI revealed cerebral atrophy (*n =* 11), ventriculomegaly (*n =* 8), white matter abnormality (*n =* 4) and corpus callosum abnormality (*n =* 14) in 64.7%, 47%, 25%, and 87.5% of patients, respectively. Two patients had flat pons.

The homozygous missense variant c.419G > A (p.Arg140His) was identified in all patients(*n =* 17). *CLP1* group was observed to spread to five regions in Turkey.

### EXOSC3

The *EXOSC3* group with PCH consisted of seven patients, five male and two female. Development delay(gross motor/fine motor/language), intellectual disability, brainstem findings(central apnea, dizziness, vertigo, impaired swallowing), cerebellar deficit(nystagmus, intentional tremor, dysmetria, scanning speech), motor deficit, gait disorder(ataxia and spastic) were reported in 100% of the patients. Half of the patients had proximal weakness, while the others consisted of tetraplegia (*n =* 2) and paraplegia (*n =* 1). Two patients revealed fasciculation. Additionaly, two patients had seizures. The onset of the seizures was defined as 2 months. Moreover, seizures were treated with monotheraphy in one patient. One of them followed up seizure free. Regression, autistic features and ADHD were described in 28.5%, 25%, and 42.8%, respectively. Poor eye contact, musculoskeletal (scoliosis, muscle atrophy) and gastrointestinal disorder (constipation, stool incontinence, feeding with percutaneous endoscopic gastrostomy tube), immunodeficiency (recurrent infections) were detected in 75%, 66.6%, 66.6%, and 3.33%, respectively. The mean age of the MRI applicationwas 6.26 ± 5.24 years. The brain MRI revealed the butterfly pattern and ventriculomegaly in one patient associated with compound heterozigot variant, c.619_622dupATTA, c.474 + 110G > A). Other abnormalities including white matter abnormality (*n =* 2), corpus callosum abnormality (*n =* 3), and cerebral atrophy (*n =* 3) in 66.6%, 100%, and 75% respectively.

Genetic analysis revealed homozygous missense variant c.572G > A in three patients, other homozygous missense variant c.395A > C in three patients, and the compound heterozigot variant c.619_622dupATTA, c.474 + 110G > A in one patient.

### AMPD2

This group included two male and four female patients. Term pregnancy, microcephaly, development delay(gross motor/fine motor/language/social),, and intellectual disability were displayed in 100% of the patients. All of the patients revealed tetraplagia, half of the patients had axial hypotonia and distal hypertonia. Seizures were identified in 66.6% of the patients. The onset of the seizures was described as 16.8 ± 14.18 months. Seizures were treated with monotheraphy in one patient and polytheraphy in three patients. Among the patients, seizures were controlled in only one patient. None of them were reported as seizure free. Only one patient had autistic features. Eye abnormality (poor eye contact), musculoskeletal (scoliosis, muscle atrophy, contractures) and genitourinary disorder(micropenis) were revealed 40%, 50% and 40% of the patients, respectively. The age of the first MRI was at 5.77 ± 1.57 years. The brain MRI revealed the dragonfly pattern in one patient (nonsense/frameshift variant, c.1110del/AMPD2(NM_001368809.2):c.1239del) and the figure of “8” of the midbrain in two patients (nonsense/frameshift variant, c.1110del/AMPD2(NM_001368809.2):c.1239del and frameshift variant, c.1652del). In addition, venticulomegaly (*n =* 3), white matter abnormality (*n =* 2), corpus callosum abnormality (*n =* 5), and cerebral atrophy (*n =* 5) were reported in 50%, 40%, 83.3%, and 83.3%, respectively.

All of the patients showed five different homozygous *AMPD2* variants: c.1110del/AMPD2(NM_001368809.2):c.1239del, c.2096A > G, c.241C > G, c.1661C > G and c.1652del (supplementary material).

### TSEN54

This group consisted of three male and three female patients. TSEN54-group characterised by term pregnancy, microcephaly, development delay, and intellectual disability with 100%. Dysmorphic features and brainstem findings (dysphagia) were described in one patient. All patients revealed motor deficit included four tetraplagia and two paraplegia. Most of the patients (83.3%) presented hypertonia. Only one patient had spastic gait disorder. Seizures were determined in 80% of the patients. The onset of the seizures was9.8 ± 15.15 months. The seizures were treated with monotheraphy in one patient and polytheraphy in three patients. One of them became seizure free. In addition, one patient presented regression. Eye abnormality (poor eye contact) and immunodeficiency (recurrent infections) were seen in 33.3%. The respiratory (stridor), endocrine disorder (hypocalcemia), musculoskeletal disorder (hip dislocation + kyphosis), genitourinary(abnormal genitalia), and gastrointestinal (constipation) disorders revealed in one patient. Only one patient had VEP/ERG abnormality. The age of first MRI was 3.04 ± 3.51 years. The brain MRI showed the dragonfly pattern (*n =* 2), venticulomegaly (*n =* 2), white matter abnormality (*n =* 1), cerebral atrophy (*n =* 1) and corpus callosum abnormality (*n =* 3) in 33.3%, 40%, 16.6%, 20%, and 60%, respectively.

All patients showed the same homozygous missense variant c.919G > T (p.A307S). The patients attended from two different regions.

### RARS2

Five patients including three male and two female joined the group. Consanguinity and term pregnancy were reported in 80%. Microcephaly, dysmorphic features and cerebellar deficits were detected in 75%, 50%, and 66.6% respectively. Development delay(gross motor/fine motor/language/social), intellectual disability, gait disorder (ataxia and spastic), eye (poor eye contact, strabismus, optic atrophy), seizures and EEG abnormalities revealed in 100% of the patients. Two patients showed signs of autism and one patient displayed regression. Motor deficits consisted of three tetraplagia, one proximal weakness and one axial hypotonia and distal hypertonia. Hypotonia occured in four of the five patients. The mean age onset of the seizures was 8.67 ± 13.27 months. The seizures were treated with monotheraphy in one patient and polytheraphy in two patients. One of them was reported as seizure free. One patient with visual abnormality (decreased visual acuity) and two patients with brainstem findings (dysphagia, central apnea, hearing deficit) were revealed. Renal (neurogenic bladder, nephrolithiasis) and gastrointestinal disorders (feeding abnormalities, feeding with percutaneous endoscopic gastrostomy tube, constipation) were described in 40% and 80%, respectively. Immunodeficiency (recurrent infections) and musculoskeletal disorder (muscle atrophy) were reported in one patient. MRI revealed cerebral atrophy in three patients (60%), ventriculomegaly in one patient (25%), white matter abnormality in two patients (50%) and corpus callosum abnormality in one patient (20%). One patient also had VEP/ERG abnormality. Three patients from two distinct regions revealed the homozygous missense variant c.1037C > T (p.Thr346). The other two variants (missense) were found c.722G > T and c.1026G > A.

### VLDLRL

Three male and two female patients were included in this group. Consanguinity, microcephaly, development delay, intellectual disability, gait disorder (ataxia), and eye abnormality (strabismus) were reported in 100%. Cerebellar deficits (nystagmus, intentional tremor, dysmetria), and paraplegia were observed in 80%. Four of them showed paraplegia and one of them revealed monoparesia. Dysmorphic features, hypertonia and musculoskeletal disorder (scoliosis) were displayed in one patient. The mean age of the first MRI was 0.58 ± years. On brain MRI, thicking cerebral cortex as pachygryria described in three patients associated with frameshift, c.1249_1255del and stop codon, c.835C > T. Three homozygous variants (c.1249_1255del, c.1970 T > A, and c.835C > T) from three different regions were counted.

### HEATR5B

Three patients of *HEATR5B* were determined with consanguinity, microcephaly, development delay, regression, intellectual disability, brainstem findings (dysphagia, central apnea), hypotonia, eye abnormality (poor eye contact, microphthalmia), respiratory (recurrent aspiration, tracheostomy) and gastrointestinal (aganglionic megacolon, gallstone, feeding with percutaneous endoscopic gastrostomy tube) disorders were observed in 100%. One patient revealed tetraplegia and also one patient had spastic gait disorder. Three patients had seizures. The onset of the seizures was defined as one month. Decreased visual acuity and nystagmus as a cerebellar sign were found in two patients. Two patients revealed immunodeficiency (recurrent infections). The age defined for the performed MRI was 0.66 years. It was described one patient (33.3%) with basal ganglia abnormality, two patients (100%) with white matter abnormality and cerebral atrophy, and three patients (100%) with ventriculomegaly and corpus callosum abnormality (hypoplasia). All of the patients came from Black sea region and showed the homozygous variant c.5051–1G > A (splice site).

### CASK

The *CASK* group consisted of three patients with term pregnancy, microcephaly, developmental delay, intellectual disability, cerebellar deficits (nystagmus), tetraplagia, dysmorphic features, and eye abnormality ( poor eye contact, optic atrophy) in 100%. Autistic features and ADHD were diagnosed in one patient. Seizures was determined in one patient. The onset of the seizures was reported as one month and treated with polytheraphy as a diagnosis of epileptic encephalopathy. One patient revealed visual abnormality including rod and cone dystrophia. Brainstem findings (dysphagia, central apnea, impaired swallowing), gait (ataxia + spastic), musculosceletal (joint stiffness, contracture, pes equinovarus) and gastrointestinal (feeding abnormality, constipation) disorders were observed in two patients. The age reported for the performed MRI was 3.91 ± 3.64 years. One of them revealed trigonocephaly. One of them also showed mega sisterna magna and white matter abnormality. The variants revealed c.477del (frameshift), c.1910G > A (missense) and c.82C > T (nonsense).

### MINPP1

The *MINPP1* group had two patients. Developmental delay, microcephaly, dysmorphic features, regression, and seizure were determined in one patient. The onset of the seizures was revealed as two months. The seizures were controlled with monotheraphy. Consanguinity, brainstem findings (dysphagia, impairing swallowing), cerebellar deficits (nystagmus), eye abnormality (cataract, ptosis) were reported in two patients. One patient had tetraplegia. Additionally, one of them displayed hypertonia and AHDH. On MRI findings, white matter and basal ganglia abnormality with two patients, ventriculomegaly, cerebral atrophy, and corpus callosum abnormality with one patient were reported. The variants showed c.74C > A(nonsense) and c.181_182insGAC (nonsense).

### EXOSC8

Two patients formed the *EXOSC8* group. Microcephaly, development delay, intellectual disability, autistic features, gait disorder (ataxia, neuropathic), and EEG abnormality were observed in two patients (100%). One patient revealed ADHD. Seizures were observed in two patients. The onset of seizures were reported as 6.5 ± 2.12 months. One patient was treated with monotherapy and other one with polytherapy. One patient was seizure free after the medication. Dysmorphic features, visual abnormality (decreased visual acuity, hemianopsia), brainstem findings (central apnea) and cerebellar deficit (scanning speech, oculomotor apraxia) were defined in one patient. Tetraplagia and hypotonia was described in one patient. Eye abnormality (strabismus, poor eye contact), respiratory (tracheostomy), musculoskeletal (scoliosis, joint stiffness, contracture), gastrointestinal disorder (feeding abnormalities, constipation) were observed in one patient. The age reported for the performed MRI was 5.5 ± 3.53 years and main finding was only PCH. Two patients had EEG abnormalities and one patient showed ENMG abnormality. Two missense variants showed c.815G > C and c.719G > C.

### TOE1

The *TOE1* group included two patients. Consanguinity, term pregnancy, microcephaly, development delay, regression, intellectual disability, cerebellar deficits, gait disorder, eye abnormality (strabismus), musculoscelatal disorder (muscle atrophy), dysmorphic features in two patients. One patient had seizures. The onset of seizures were reported as 48 months. The polytherapy was used to treat the daily seizures. Tetraplegia, paraplegia and hypertonia were observed one, one and two patients, respectively. The mean for first MRI was 2.25 ± 0.35 years. MRI showed ventriculomegaly, cerebral atrophy and corpus callosum hypoplasia in two patients. Only one variant consisted of c.572A > G (missense) was determined.

### TBC1D23

The *TBC1D23* group consisted of two patients from the same center. Consanguinity, term pregnancy, microcephaly, development delay, intellectual disability, ADHD, brainstem findings (vertigo, dysphagia, dizziness), cerebellar deficits (nystagmus, dysmetria), hypotonia, gait disorder, eye abnormality (cataract, astigmatism), cardiovascular (atrial septal defect), endocrine (hypothyroidism), musculoscelatal disorders (scoliosis), dysmorphic features, seizures (controlled with monotherapy), and EEG abnormality were reported in two patients. One patient showed autistic features. The onset of seizures were defined as 24 ± 16.97 months. The time of the MRI was reported as 4.0 ± 1.41 years. The ventriculomegaly, and corpus callosum hypoplasia were detected in both of them. The frameshift variant revealed c.743_744del.

### TSEN2

A patient with *TSEN2* (c.1091C > G) was described by consanguinity, term pregnancy, microcephaly, development delay, intellectual disability, ADHD, and tetraplagia. There was no reported any findings except for pontocerebelar hypoplasia. The missense variant was revealed c.1091C > G.

### SEPSECS

A patient carrying a homozygous noncoding variant in the *SEPSECS* (c.114 + 3A > G) gene presented with term pregnancy, microcephaly, development delay, paraplagia, hypertonia, and spastic gait disorder. There was no reported any supratentorial or infratentorial findings except pontocerebelar hypoplasia.

### CHMP1A

One patient with *CHMP1A* (frameshift, c.128_129dup) displayed with consanguinity, microcephaly, development delay, regression, seizure, intellectual disability, brainstem findings (hyperacusis, ptosis), cerebellar deficits (nystagmus, intentional tremor), visual abnormality (decreased visual acuity, blurry vision), tetraplagia, axial hypotonia and distal hypertonia, spastic gait disorder, cardiovascular (patent ductus arteriosus, patent foramen ovale), musculoskeletal (pes ecinovarus) findings, EEG, and VEP/ERG abnormalities. The time of the first MRI was 2 years. The ventriculomegaly, white matter abnormality, and corpus callosum hypoplasia were reported on MRI.

### PCLO

Microcephaly, development delay, intellectual disability, brainstem finding (dysphagia), tetraplagia, axial hypotonia and distal hypertonia, musculoskeletal (scoliosis, muscle atrophy, contractures), gastrointestinal (feeding abnormality) and skin (hyperpigmented spot), and ventriculomegaly on MRI were determined in one patient with *PCLO* (insersion, c.8776_8777insATG).

## Discussion

We described a wide range of clinical, laboratory, and neuroimaging manifastations of 64 patients with PCH based on genetic analysis. In this study, we detected the most common mutation was *CLP1* gene. In the literature, Namavar et al. reported that the most common mutation was *TSEN54* gene(*n =* 88), including 106 patients who tested positive for the *TSEN2, 34, 54* or *RARS2* gene [3]. Recently, Nuovo et al. reported that most common causative gene was *CASK,* in 43 pathogenic or likely pathogenic variants among 56 PCH probands[2].

*CLP1* is an RNA kinase involved in tRNA splicing and maturation. Mutation is associated with functionally impaired kinase activity and subsequent defective tRNA processing. Proposed pathogenic mechanism include intronic RNA fragment accumulation [16]. An accumulation of PCH10-causing tyrosine pre-tRNA fragments in modeling a human *CLP1* mutation (R140H) in the mouse and suppression of premature transcription termination resulting in diminished mRNA isoform variety and neurodegeneration [17, 18].

PCH10 was described simultaneously and independently, by Karaca E, et al. idendified the same homozygous rare variant R140H of the *CLP1* gene (c.419G > A; p.(Arg140His), in 11 individuals from five families [19]. Schaffer AE, et al. and Wafic M, et al. described two patient from family of Turkish origin the same mutation [20, 21]. In our study, we identified same gene mutation from 17 patients.

Clinical manifestations in PCH10 mainly occur of microcephaly, intellectual disability, severe development delay, seizure, progressive spasticity, facial dysmorphism, axonal neuropathy, thin corpus callosum, cortical dysgenesis/atrophy, ventricular dilation, delayed myelination, and white matter changes on the brain MRI [1, 19, 22]. Axonal neuropathy and cortical dysgenesis are important findings in PCH10 [19]. In the present study, four ENMG findings were found axonal sensorimotor/motor neuropathy and 11 patients revealed thick cerebral cortex. Although PCH10 has merely showed in children of Eastern Turkish region until recent years, Amin et al. reported a new family diagnosed PCH 10 from Sudan with the same founder mutation in *CLP1* gene and similar clinical and radiological features in the previous Turkish studies [7, 22]. Moreover, we exhibited compatible manifestations according to pevious reports. our study had a higher patient participitation from the Mediterranean region among the patients collected from five different regions compared to previous report including five families from Eastern Turkey [19].

EXOSC protein consists of nine core proteins (EXOSC1-9) affect the exosome. The substantial task of the exome includes processing of mRNA, rRNA and probably tRNA of RNA precursor, transcript and unspliced degradation, and regulation of RNA processing in cerebellar and spinal neurons [9, 23–25]. Müller et al. emphasize the connection between exosome function, ribosome biogenesis, p53-dependent signalling about pathogenesis of exosome-related disorders (EXOSC3, EXOSC8, EXOSC9) [23]. In the literature, *EXOSC3* mutations have constituted in nearly half of PCH1 cases [24].

*EXOSC3 (PCH1B)* mutations can cause from mild to severe phenotypic spectrum including central and peripheral motor dysfunctions associated with anterior horn cell degeneration, muscle weakness, hypotonia, respiratory insuffi ciency and congenital contractures [25]. In this study, about half of patients with *EXOSC3* mutations had the most commonly reported pathogenic variant, c.395A > C, p.(Asp132Ala) and about the other half had c.572G > A, p.(Gly191Asp). One patient with a compound heterozigot variant c.619_622dupATTA, c.474 + 110G > A had also butterfly pattern. It is suggested that homozygosity for c.395A > C, p.(Asp132Ala) variant exhibits a milder clinical picture than compound heterozygosity [9, 26]. The phenotype of the patients with both c.395A > C and c.572G > A, p.(Gly191Asp) can be interpreted as moderate. In our study, similar to literature, we determined the homozygous missense variant c.572G > A in three patients, other homozygous missense variant c.395A > C in three patients, and the compound heterozigous variant c.619_622dupATTA, c.474 + 110G > A in one patient. Based on the clinical picture of our patients, we recommend that homozygous missense variants are milder than compound heterozygous variant, in line with the literature.

Mutations of *EXOSC8* lead to PCH with central nervous system demyelination PCH1C [22, 27]. Clinical features include psychomotor deficit, cerebellar and corpus callosum hypoplasia, hypomyelination, respiratory failure, contractures, spinal muscular atrophy, and hearing impairment [7, 27]. Unlike the literature, our two patients exhibited microcephaly, seizures, autistic features, gait disorder (ataxia, neuropathic), and EEG abnormality one of them also showed severe motor axonal neuropathy on EMG.

PCH9 is caused by bi-allelic mutations in the *AMPD2* gene which transforms adenosine monophosphate to inosine monophosphate associated the purine metabolism leads to neurotoxicity due to intracellular adenosine accumulation and derived nucleotids [1, 28, 29]. *AMPD2* is expressed primarily in Purkinje cells within the cerebellum and causes severe cerebellum involvement [28]. In our study, severe clinical profile revealed as all patients had tetraplagia in *AMPD2*-group. However, it was also attractive half of the patients had axial hypotonia and distal hypertonia. Moreover, approximately half of the patients showed cortical visual impairment as noted ‘poor eye contact’. On MRI findings, one patient displayed dragonfly pattern (comparatively preserved vermis and atrophic cerebellar hemispheres) and two patients with abnormal midbrain describing a figure of “8” which is accepted typical imaging findings for PCH9. In addition, corpus callosum abnormalities (thin/absent) were detected higher rate than in series of Scola and friends as in our patients (hypoplasia/agenesis, 83.3%) [30]. We reported five different homozygous *AMPD2* variants in six patients. Therefore, we can not establish clear genotype–phenotype correlation like the recently reported study [28].

Mutations in all subunits of *TSEN* lead to diverse types of PCH ( Type 2A,2B, 2C and 2F, and PCH4, PCH5) [17]. The most prevelant type is PCH2A caused by mutations A307S variant of *TSEN54* gene. Ultimately, *TSEN* complex occur of two catalytic (*TSEN2* and *TSEN34*) and two structural (*TSEN15* and *TSEN54*) protein subunits. *TSEN54* constitudes a component of the transfer-RNA (tRNA) splicing endonuclease complex. *TSEN2, TSEN34* and *TSEN15* mutations are classified as PCH2B, PCH2C and PCH2F, respectively. These subtypes infrequently exist and share similar clinical features [1, 3, 31]. The dystonia/chorea, impaired swallowing, central visual impairment, variable degrees of spasticity, recurrent infections, apneas, thermoregulation and sleeping disorders can be counted as distinguishing features from common features in PCH [1, 3, 7]. The dragonfly-like cerebellar pattern on MRI is often associated with homozygous p.A307S mutation [3, 30]. The dragonfly pattern was revealed in two patients in the present study, too. In our study, all six patients with this mutation were classified as PCH2A, consistent with clinical findings in the literature.

TSEN2 is one of the protein subunit within TSEN complex. TSEN2 mutations, although infrequent, result in PCH2B characterized by common neurological findings including microcephaly, developmental delay, intellectual disability, epilepsy, dyskinesia, central visual impairment, and hyperkinetic involuntary movements [7, 32]. Due to this mutation decline in protein translation via distrupted tRNA splicing occur [1]. In our study, we identified one patient with PCH2B, consistent with clinical findings in the literature.

PCH6 is resulted in biallele mutations in *RARS2*, which encodes mitochondrial arginyl-tRNA synthetase [33, 34]. *RARS2* is liable for simplifying the particular connection of Arginine to its cognate mitochondrial tRNA by mitochondrial translation [1, 34]. Distinctive features occur of hypotonia, refractory epilepsy, lactic acidosis, and/or defective activity of mitochondrial respiratory chain, impaired swallowing, dysconjugate eye movements, optic atrophy, visual impairment, apneic episodes, and edema of extremities [3, 7, 33–35]. On half of our patients, we described white matter abnormality on MRI and dysmorphic features. In our cases, autistic features, renal disorders (neurogenic bladder, nephrolithiasis) are distinctive features from the other reported patients in the literature.

The loss-of-function mutations in the multiple inositol-polyphosphatephosphatase 1 gene (*MINPP1*) cause PCH16 by impairing neuronal differentiation and then growing cell death through induced stem cells via elevated inositol hexakisphosphate (IP6) producing increased chelation of intracellular cations [14]. Ucuncu et al. reported the ptosis/cataract was reported as a striking finding in PCH16 patients from other PCH subtypes [14]. We also in two patients with *MINPP1* mutations. On MRI findings, Ucuncu et al. and Appelhof et al. underlined basal ganglia hypoplasia/atrophy [14, 36]. Similarly, our two patients revealed the ptosis, cataract and the basal ganglia hypoplasia/atrophy (One of them took place in report of Ucuncu et al.[14]).

The target of EGR1 protein 1 (*TOE1*) plays a crucial role various biological processes including sustaining genome stability by the maturation of the RNA pattern, cell cycle regulation, and the maturation of a variety of small nuclear RNAs (snRNAs) [37]. To date, 17 patients with *TOE1* mutations were reported. Among these patients, twelve of them revealed the ambiguous genitalia [12, 37, 38]. We described two patients with PCH7, one exhibiting ambiguous genitalia, and in both of them observed regression, cerebellar deficits, strabismus, and dysmorphic features are different features from literature.

TBC1D23 protein was defined as a vesicle-Golgi adaptor [39].The pathogenic modifications of TBC1D23 cause the deterioration of vesicle trafficking, resulting in an aberrant neuronal growth and brain development in zebrafish. The mechanisms decline number and size of neurite outgrowth in TBC1D23-deficient human fibroblast and primary neurons, and also downregulation of TBC1D23 influencing cortical neuron positioning and causing primary cortical lamination anomalies in the mouse cortex [40, 41]. TBC1D23 deficiency due to homozygous mutations in *TBC1D23* gene leads to PCH1 [1, 7, 39, 41]. In our study, Two patients with *TBC1D23* de novo frameshift mutations exhibited controlled seizure, brainstem findings (vertigo, dysphagia, dizziness), gait disorder (ataxia and spastic), cardiovascular (atrial septal defect), hypothyroidism, and scoliosis which were not typically reported in the literature.

*SEPSECS* gene encodes the SepSecS protein which catalyzes the final step of Sec biosynthesis that play a crucial role in brain development of mammals. [42]. PCH2D caused by *SEPSECS* mutations defined as contractures, sleep disturbances, irritability, edema of face and limbs, polyneuropathy, optic atrophy, microcephaly, epilepsy, nystagmus, and intellectual disability [7, 32]. In this study, only one patient was described compatible with clinical findings in the literature.

Chromatin modelling and cell proliferatioN (*CHMP1A*) is assumed a rare mechanism associated with PCH [1]. Clinical phenotype includes abnormal muscle tone, dystonia, ataxia, cortical visual impairment, and choreiform movements [1, 7]. Our study described only one patient with *CHMP1A* (PCH8) mutations showing regression, seizure, brainstem findings (hyperacusis, ptosis), cerebellar deficits (nystagmus, intentional tremor), ventriculomegaly, white matter abnormality, cardiovascular (patent ductus arteriosus, patent foramen ovale), EEG and VEP/ERG abnormalities, which differed from the literature.

PCH3 is caused by mutations in the *PCLO* gene and clinical features exist of facial dysmorphism, optic atrophy, cerebellar atrophy, and neonatal hypotonia. The pathogenic mechanism is loss of piccolo affecting the disruption of various synaptic proteins and the separate of the synapse. Piccolo is encodes by *PCLO* and responsible for synaptic vesicle organization, persistence of synaptic unity, organizing presynaptic ubiquitination and proteostasis [1, 7]. One patient with PCH3 revealed clinical findings similar to previously reported case. Although Ahmet et al. reported homozygous, nonsense variant in the *PCLO* gene, we report a heterogeneous insersion variant (c.8776_8777insATG, p.Asp2926dup) [43].

*VLDLR*–associated PCH is a rare autosomal recessive disorder. The *VLDLR* protein is responsible for a sunstantial part of fatty acid metabolism and it plays a prominent role in the reelin signaling pathway [44]. Reelin composes neuronal positioning in the cortical brain structures and the migration of neurons along the radial glial fiber network [45]. The patients clinically presents with nonprogressive cerebellar ataxia, delayed ambulation, quadripedal gait, varying degrees mental retardation, dysarthria, cerebellar hypoplasia and less frequentl seizures, strabismus, short stature, pes planus and dysmorphic features [1, 15]. Simplified gyration of the cerebral hemispheres, minimally thickened cortex, and absance anteroposterior gradients and tiny brain stem including especially the pons are characteristic MRI findings. We identified five patients with three homozygous variants characterised by ataxia, strabismus and cerebellar deficits. Two of them revealed bipedal gait similar to literature [46]. Moreover, three of them with pachygyria on MRI similar to the patients were reported [47].

*HEATR5B* protein play a substantial role in endocytosis and membrane trafficking [48, 49]. Glosh et al. reported four patients from two families showing PCH with seizures, severe intellectual disability, and motor delay. Both families revealed homozygous variants neighboring to canonical splice sites (c.5051–1G > A and c.5050 + 4A > G) in *HEATR5B* gene. The family consisting of two siblings carrying the homozygous variant c.5051–1G > A was also included in this study [13]. Our study identified three patients with PCH carrying the same homozygous variant (c.5051–1G > A) in the HEATR5B gene. MRI findings were remarkable and ranged from ventriculomegaly to white matter, basal ganglia, and corpus callosum abnormalities in all patients. Additionally, cortical visual impairment and microphthalmia, respiratory and gastrointestinal disorders were considerable and common features in all patients. [13].

The *CASK* protein is connected to the membrane-associated guanylate kinase family and comprises various functional domains [50]. *CASK* protein unites multiprotein complexes and also responsible for synaptic interaction, protein trafficking, signaling of ion channels, and arrangement of gene expression throughout neural development [51, 52]. Loss of function *CASK* variants exhibit most severe phenotypic manifestations present in females, with in utero death in males [2, 52]. Despite of the divergent phenotypic spectrum mutations in the *CASK* gene, it can present facial dysmorphism, sensorineural hearing loss, optic atrophy, retinopathy, hypohidrosis, microcephaly, developmental delay, limb hypertonia, pronounced cerebellar hypoplasia, various extent of pons hypoplasia, and a normal-sized corpus callosum [7, 35, 52]. All our patients shared similar clinical and neuroimaging findings in the literature.

However, it is necessary to consider that the current study has some limitations. One of them is the method of the study is retrospective. The second is only accessible data was recorded.

The phenotypic spectrum of PCH is much broader than more than anticipated. This multicenter study has the feature of being the most comprehensive study conducted in Turkey on PCH. Although the most common causative gene was defined as *CASK* or *TSEN54* in a few large cohort studies in the literature, in our study; *CLP1* mutation (c.419G > A, p.Arg140His) was described the most common causative gene in Turkey. Further investigations and large cohort of patients are need to determine the clear genotype–phenotype correlations.

## Supplementary Information

Below is the link to the electronic supplementary material.

Supplementary file1 (XLSX 32 KB)

## Data Availability

No datasets were generated or analysed during the current study.
